# Supplementary material for: Association between oral microbiome and seven types of cancers in East Asian population: a two-sample Mendelian randomization analysis
Source: Front Mol Biosci. 2023 Nov 21;10:1327893. doi: 10.3389/fmolb.2023.1327893 (PMC10702768; doi:10.3389/fmolb.2023.1327893)
Supplement: Supplementary file 1 [file DataSheet1.ZIP › Supplementary Materials/MR plots of saliva/saliva═╝/Pancreatic cancer/pheno.710_to_pancreatic cancer_forest.pdf]

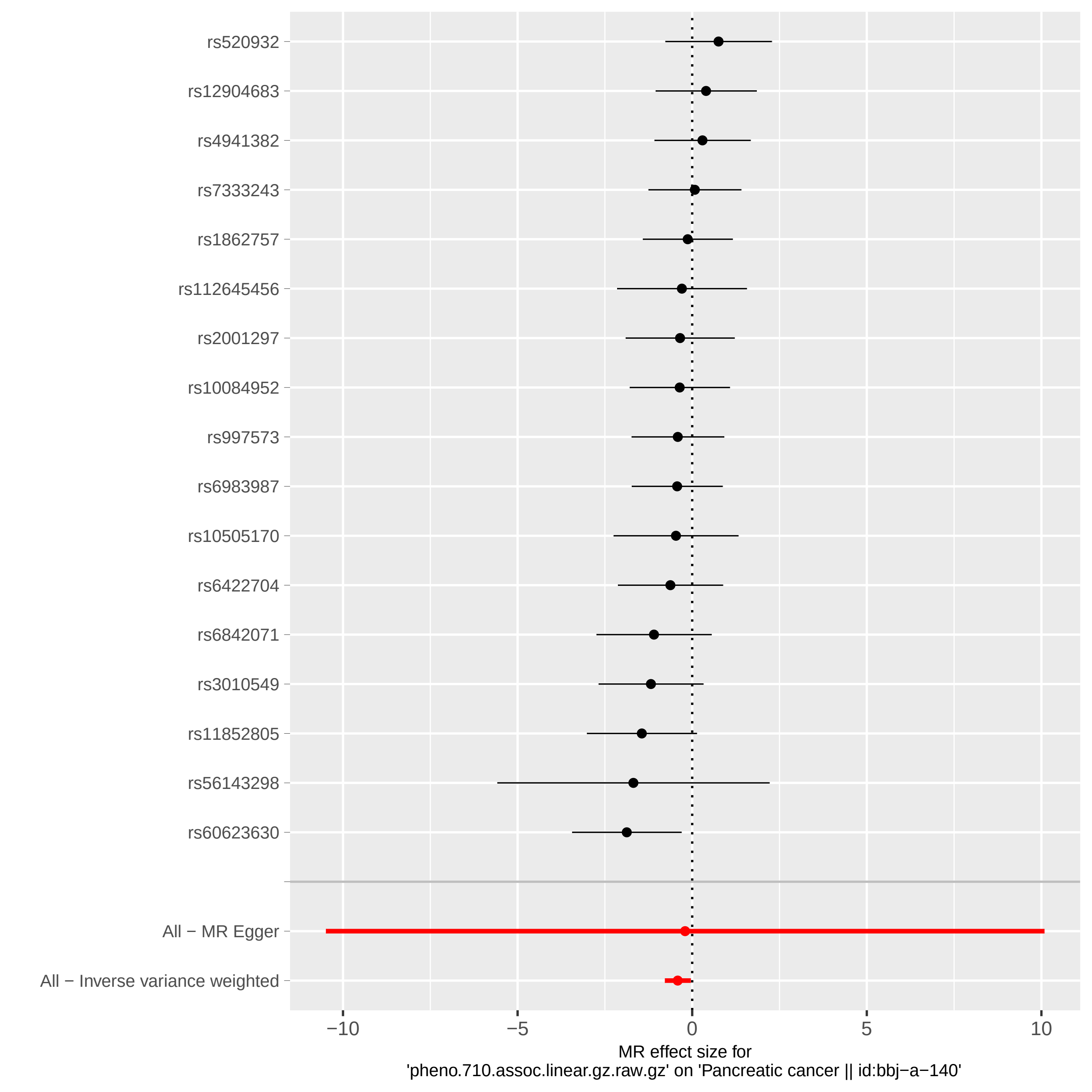
rs520932
rs12904683
rs4941382
rs7333243
rs1862757
rs112645456
rs2001297
rs10084952
rs997573
rs6983987
rs10505170
rs6422704
rs6842071
rs3010549
rs11852805
rs56143298
rs60623630
All − MR Egger
All − Inverse variance weighted
−10
−5
0
5
10
MR effect size for
'pheno.710.assoc.linear.gz.raw.gz' on 'Pancreatic cancer || id:bbj−a−140'
